# Course Patterns of the Marginal Mandibular Branch of the Facial Nerve over the Facial Vessels in Relation to the Inferior Mandibular Border

**DOI:** 10.3390/diagnostics16172762

**Published:** 2026-08-28

**Authors:** Alexandra Diana Vrapciu, Mugurel Constantin Rusu

**Affiliations:** Division of Anatomy, Faculty of Dental Medicine, “Carol Davila” University of Medicine and Pharmacy, 050474 Bucharest, Romania; mugurel.rusu@umfcd.ro

**Keywords:** marginal mandibular branch of the facial nerve, facial nerve, inferior border of the mandible, facial artery, surgical anatomy, branching pattern, anastomosis, submandibular surgery, rhytidectomy

## Abstract

The marginal mandibular branch of the facial nerve (MMB) is a surgically relevant extracranial branch of cranial nerve VII, vulnerable during submandibular, parotid, and cervicofacial procedures. Its relationship to the facial vessels and the inferior border of the mandible (IBM) is variable and has been studied for more than six decades. This review analyses the course of the MMB as it crosses the facial artery and vein, focusing on its vertical position relative to the IBM, together with branching patterns, fascial relationships, anastomoses, landmarks, injury, and recovery. The MMB usually crosses the facial vessels superficially, but deep passage and looping have been reported. Its vertical position is segment-dependent: posterior to the facial artery, one or more branches may descend below the IBM in approximately 19–39% of cases, whereas anterior to the artery the nerve is almost always above it. A meta-analysis of 1861 hemifaces reported a pooled prevalence of 39% for at least one branch below the IBM. Reported iatrogenic injury rates range from 16% to 47% (largely transient), and recovery is variable, reflecting a richer communicating network than once assumed. The facial artery crossing at the IBM is a practical landmark for locating the MMB. The traditional 2 cm rule does not reliably protect the nerve, which may descend below this level, especially posterior to the vessels and during neck extension. Safer access depends on combined bony and vascular landmarks, subplatysmal dissection, and assessment of branching variability.

## 1. Introduction

The marginal mandibular branch of the facial nerve (MMB) is a terminal motor branch of cranial nerve VII (CN VII). It leaves the antero-inferior border of the parotid gland. It passes forwards through the submandibular region to the depressor anguli oris (DAO), depressor labii inferioris (DLI), mentalis, and lower fibres of orbicularis oris [1,2,3]. Additional reported targets include the risorius and the lower part of the buccinator [4,5]. Together, these muscles mediate lower-lip depression, oral continence, phonation, and lower facial expression.

MMB injury is a recognised complication of surgery in the submandibular triangle and parotid region. Reported rates are 0–20% after submandibular gland excision, 5.6–16.3% after parotidectomy, and up to 23% after neck dissection [6,7,8]. The deficit causes asymmetric lower-lip depression, flattening or inversion of the lip, deviation of the mouth angle, drooling, and impaired phonation, and is most evident during smiling or crying [9,10].

Prevention of MMB injury depends on recognising its variable course, especially its vertical relationship to the inferior border of the mandible (IBM) and its topography relative to the facial artery (FA) and facial vein (FV). Both vessels serve as operative landmarks [9,11]. Since the study by Dingman and Grabb (1962) [12], reported patterns have remained variable, partly due to population differences, study methods, and nerve displacement during surgical positioning.

The most comprehensive quantitative synthesis to date is the systematic review and meta-analysis by Marcuzzo et al. (2020), which pooled data from 28 studies and 1861 hemifaces and established a pooled prevalence of 39% for at least one MMB branch below the IBM [13]. However, the meta-analytic format carries inherent structural constraints that limit its translational scope. It can pool only outcomes that multiple studies report in directly comparable formats: qualitative pathway classifications, segment-by-segment positional mapping, and needle-traced courses cannot be reduced to poolable metrics. Methodologically discordant dissection techniques—standard anatomical dissection versus microanatomical dissection at higher magnification—produce systematically divergent anastomotic frequencies that pooling conflates rather than resolves, as reflected in the extreme heterogeneity (I^2^ ≥ 93%) reported across virtually all outcomes by Marcuzzo et al. themselves. Study designs based on radiological morphometry, intraoperative electrostimulation, and microneuroanatomical mapping fall outside the eligibility criteria of anatomical meta-analyses but are essential for a complete functional and surgical picture. Finally, mechanistic and clinical content—fascial plane transitions, functional innervation hierarchies, recovery biology, repair principles, and the proximity of the MMB to the mandibular ligament—is not produced by pooled prevalence estimates. The present narrative review addresses these structural gaps and integrates a substantial body of evidence published after Marcuzzo et al.’s search cutoff.

This review collates anatomical evidence on the MMB in relation to the facial vessels and the IBM, including branching, fascial planes, nerve dimensions, landmarks, injury, recovery, and surgical access.

### Literature Search and Terminology

This narrative review was informed by a search of PubMed/MEDLINE, Scopus, and Web of Science from database inception to March 2026. Search terms combined ‘marginal mandibular branch’, ‘marginal mandibular nerve’, ‘facial nerve’, ‘inferior border of the mandible’, ‘mandibular angle’, ‘facial artery’, ‘facial vein’, and ‘surgical anatomy’. We screened the reference lists of retrieved articles for additional sources. Anatomical (dissection and plastination), radiological and morphometric, intraoperative, electrostimulation, and microneuroanatomical studies addressing the topography, branching, vascular relationships, innervation, injury, or repair of the MMB were eligible; no language restriction was applied, and—consistent with the narrative format—no formal risk-of-bias scoring or quantitative pooling was undertaken. Anatomical terminology follows Terminologia Anatomica [14].

## 2. Fascial Planes, Depth, and General Course of the MMB

After leaving the antero-inferior border of the parotid gland, the MMB passes forward in the neck. It lies deep to the platysma and superficial to the investing (superficial) layer of the cervical fascia, within the subplatysmal plane [7,8,15]. Between the parotid gland and the facial vessels, it is close to the submandibular gland and perifacial level IB lymph nodes.

At the anteroinferior border of the masseter, the nerve usually crosses laterally to the FA and FV [1,9,16]. This relationship is reported in 83-98% of dissected hemifaces [16,17,18]. After the vascular crossing, the MMB ascends over the mandibular body and divides into terminal branches in the lower face.

The nerve’s depth varies along its course and relative to the IBM. Branches above the mandibular border have been reported deep to the superficial layer of the parotid fascia, whereas branches below the border may be intrafascial [19]. At the level of the facial vessels, the nerve is fixed by deep fascia to the masseter and mandible [20]. A transition zone, where the MMB exits the parotid-masseteric fascia and enters the subplatysmal plane, has been described 23.1 mm from the gonial angle along the inferior mandibular margin [21]. Over and anterior to the masseter, the nerve lies beneath a thin superficial musculo-aponeurotic system (SMAS) layer, which increases its exposure during cervicofacial and aesthetic dissection [19].

These plane changes have practical consequences during dissection. Posterior to the facial vessels, subplatysmal dissection, performed superficial to the cervical fascia, leaves a protective tissue bridge over the nerve. Anterior to the vessels, the nerve lies within the platysma-SMAS layer and is more exposed during superficial dissection [8].

The mandibular ligament is also relevant in this region. Minelli et al. (2023b) [22], studying 49 anatomical specimen heads using layered dissection, histology, sheet plastination, and micro-computed tomography, showed that the true mandibular ligament lies only in the deep subplatysmal plane and is formed by the combined attachment of the platysma, DLI, and DAO to the mandible. The main MMB branch passes 1–2 mm from this ligament before continuing cephalad towards the mentalis, remaining deep to the platysma, DAO, and DLI. This distance is smaller than the 9.7 mm reported in studies measuring from a subcutaneous ‘mandibular ligament’. A deep fat layer over the mandible, between the masseter and the mandibular ligament, contained the FV posteriorly, the FA anteriorly, and the MMB crossing both vessels superficially. Accordingly, deep release of the mandibular ligament during rhytidectomy places the MMB at risk and should be avoided [22].

## 3. Nerve Length and Dimensions

Direct measurements of MMB length are uncommon, although they define the nerve segment exposed in the operative field. In a fresh anatomical study, the mean (standard deviation [SD]) total length from origin to muscular termination was 33.57 (3.41) mm on the right and 33.51 (4.88) mm on the left [21]. Thus, most of the exposed nerve length lies within the submandibular segment.

Sindel et al. (2021) [23] mapped the MMB along the inferior mandibular border by placing needles at 5 mm intervals from the gonial angle to the muscular termination point in 12 fresh anatomical specimens. The highest positions were 6.9 mm above the mandibular base on the right and 6.5 mm on the left; the lowest positions were 4 mm below the base on the right and 3 mm on the left. Significant left-right differences occurred in the middle portion of the course, 10–25 mm from the gonial angle. The terminal point was at the DAO, opposite the second premolar teeth. The authors proposed the inferior mandibular border as the primary reference for tracing the nerve because soft-tissue landmarks, such as the FA, are less stable [23].

Terminally, the MMB divides into fine intramuscular branches. Termination most often occurred as multiple small twigs entering the DAO, DLI, and mentalis (88%) [1]. Other authors reported that termination was deep within the ipsilateral lower-lip muscles in all specimens [4].

## 4. Relationship to the Inferior Border of the Mandible

The vertical position of the MMB relative to the IBM is the anatomical parameter with the most direct surgical consequence, as it determines the safe depth of submandibular incisions and the risk posed by subplatysmal dissection in the gonion-to-facial-artery segment. Reported findings vary substantially between studies, reflecting differences in specimen type (embalmed versus fresh anatomical specimen versus intraoperative), neck position, dissection methodology, and the segment of the course examined relative to the FA. Table 1 summarises the principal studies. The subsections below examine these data in sequence, from the foundational anatomical paradigm established by Dingman and Grabb (1962) [12] through subsequent corroborating and divergent anatomical series, pooled meta-analytic estimates, and the effect of intraoperative neck positioning on nerve level.

### 4.1. The Dingman and Grabb Paradigm

Dingman and Grabb (1962), in 100 facial halves, found that the MMB was above the IBM in 81% of specimens when posterior to the FA; in the remaining 19%, one or more rami formed a downward arc reaching up to 1.0 cm below the IBM. Anterior to the FA, the nerve was above the IBM in all specimens [12]. This anterior–posterior distinction remains central to the surgical anatomy of the MMB.

### 4.2. Corroborating and Divergent Findings

Subsequent studies have both confirmed and modified this scheme. Wang et al. (1991) found the nerve above the IBM posterior to the FA in 67% of specimens and below it in 33% [24]. Baker and Conley (1979), based on parotidectomy experience, noted that the MMB often lies 1-2 cm below the IBM intraoperatively, illustrating the difference between anatomical and operative observations [29].

Batra et al. (2010) found the MMB was always above the IBM anterior to the FA, along the IBM in 52%, posterior to the artery, and below in 32% (above it in the remaining 16%), with a maximum inferior excursion of 1.6 cm at the angle and 1.4 cm at the body [1]. Karapinar et al. (2013) found the MMB above the IBM in all 44 hemifaces (100%), the nerve lying a mean of 21.91 mm above the border (SD 8.23; range 13.06–40.08 mm) [18]. Anthony et al. (2018) documented a mean maximum deviation of 7.12 ± 2.97 mm below the IBM, with no statistically significant left–right difference [15].

Yang et al. (2016) [30] found the MMB within 5 mm of the gonion in 82.8% of hemifaces and within 10 mm of the FA-mandible intersection in 89.7%; only small offshoots descended more than 2 cm below the mandible. They proposed safe zones of at least 2 cm below the mandible and approximately 4.5 cm anterior to the gonion [30]. Lindsey et al. (2023) [31] mapped the ‘cervical line’ in three dimensions. They supported SMAS-platysma dissection above a line from 5 cm below the mandibular angle to the facial vessel crossing, with no injury to the MMB or cervical branches [31].

### 4.3. Meta-Analytic Evidence

Marcuzzo et al. (2020), in a systematic review and meta-analysis of 28 studies and 1861 hemifaces, reported a pooled prevalence of 39% (95% confidence interval [CI]: 30–50%) for at least one MMB branch below the IBM [13]. Gatti et al. (2025), analysing 511 hemifaces from 8 studies, found the MMB at or below the IBM in 358 cases (70%) and above it in 153 (30%); the mean superior position was 1.61 cm, and the mean inferior position was 2.53 cm below the IBM [27].

### 4.4. Effect of Neck Positioning

Neck position can alter the nerve’s operative level. In a clinical series of 96 neck dissections, the MMB was below the IBM in 80% of cases, with a mean position of 2.57 mm (right) and 3.53 mm (left) below the IBM; in all but three necks, the distance did not exceed 10 mm [28]. During neck extension, the nerve is displaced inferiorly to a mean lowest point of 1.25 ± 0.7 cm below the mandible, between the anterior and posterior FVs [6].

## 5. Relationship to the Facial Vessels

### 5.1. Relationship to the Facial Artery

The MMB usually crosses the FA on its lateral (superficial) side. Karapinar et al. (2013) found all branches lateral to the FA in 97.7% (43/44) of specimens; in one case, two branches passed between the artery and vein [18]. Khanfour and El-Sayed (2014) reported at least one branch superficial to both vessels in 83.3% of 30 hemifaces [16]. Other studies found more variation: Basar et al. (1997) described passage superficial to the FA in 42%, deep in 4%, and both superficial and deep in 54% of facial halves [17]. At the ramus level, 55 of 59 individual rami (93.2%) crossed externally, whereas 4 (6.8%) divided and reunited around the vessels, forming a loop relevant to pedicle dissection [4].

Savary et al. (1997) described several marginal branches forming a neural plexus around the FA, with some passing deep to the artery while remaining superficial to the vein [32]. Touré et al. (2004), in 54 dissections, likewise found the marginal branch lateral to the facial vessels in 51 of 54 specimens (94%) but medial to the artery or vein in three [33]. The practical rule that the MMB is ‘always superficial’ is therefore an approximation rather than an anatomical constant. Table 2 summarises the principal findings on MMB–facial vessel relationships across selected studies.

### 5.2. Premasseteric Branch of the Facial Artery and Proximity to the MMB

**Figure 1:**
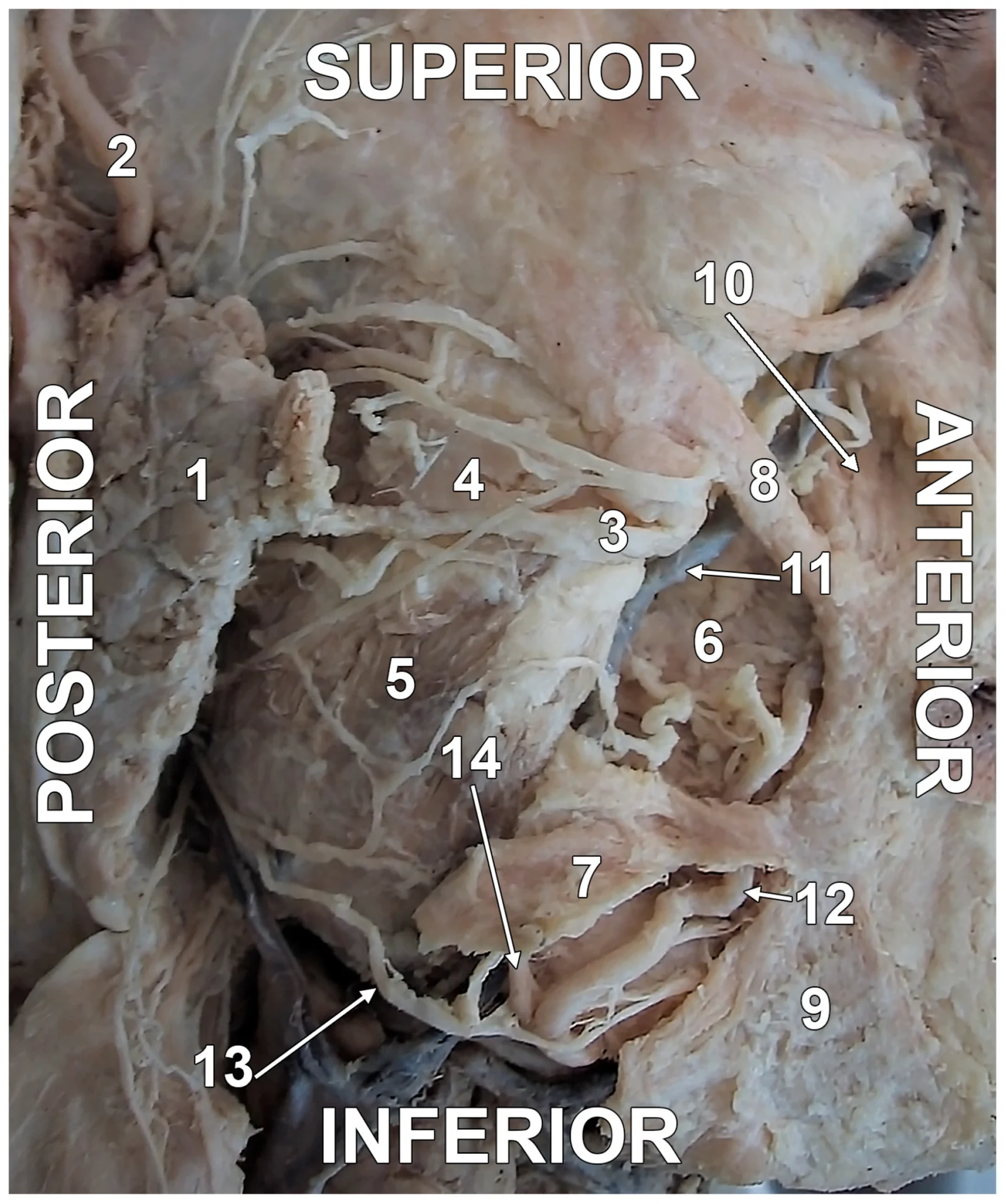
Dissection of the right hemiface depicting the neurovascular relations in the premasseteric region. Lateral view. 1. parotid gland; 2. superficial temporal artery; 3. parotid duct; 4. accessory parotid gland; 5. masseter muscle; 6. buccinator muscle; 7. risorius muscle; 8. major zygomaticus muscle; 9. depressor anguli oris muscle; 10. levator anguli oris muscle; 11. facial vein; 12. facial artery; 13. marginal mandibular branch of the facial nerve; 14. premasseteric branch of the facial artery.

The premasseteric branch of the FA (ramus premassetericus) adds a vascular structure along the anterior border of the masseter muscle, within a corridor also crossed by MMB rami. Mağden et al. (2009) [34], using microdissection under 4× magnification in 27 hemifaces, found this branch to be constant, arising separately from the FA in all specimens. Its origin lies 11.25 mm above the inferior mandibular border and 9.44 mm below the superior edge of the mandibular body; its mean diameter at origin is 1.12 mm (range 0.60–2.10 mm), and in 3% of cases its calibre equals or exceeds that of the FA trunk [34]. Mağden et al. explicitly measured the distance between the premasseteric branch origin and the MMB, finding a mean of 9.23 mm (range 3–49 mm) [34]. The minimum separation of 3 mm in that series demonstrates that these two structures can occupy the same anatomical corridor with a clinically negligible interval. This proximity is particularly relevant in high-arc MMB courses. In the Type II pathway described by Baur et al. (2014), the nerve remains consistently superior to the inferior mandibular border along its entire trajectory; Balagopal et al. (2012) documented the highest individual MMB position at the FA crossing as 5 mm above the inferior mandibular border, and Sindel et al. (2021) recorded the highest needle-mapped position at 6.9 mm above the mandibular base [9,23,25]. As the MMB ascends anteriorly from the FA crossing towards the labial commissure—reaching approximately 10.9 mm above the inferior mandibular border at that terminal point [25]—its path traverses the elevation at which the premasseteric branch originates (11.25 mm above the mandibular base) and along which that branch ascends the anterior masseteric border. In high-arc variants, this convergence occurs while the MMB is still within the masseteric region, making a crossing or near-crossing of the two structures anatomically plausible. Dissection studies have not documented whether the MMB passes superficial to or deep to the premasseteric branch at this level, leaving this point unresolved in the region’s topographic anatomy. The present dissection (Figure 1) appears to show a superficial course of the MMB over both the FA and its premasseteric branch. In practice, the premasseteric branch should be anticipated as a possible haemorrhagic source close to the MMB during mandibular angle osteotomies, correction of masseteric hypertrophy, and deep dissection at the masseteric–buccal junction.

### 5.3. Relationship to the Facial Vein

The FV is a useful landmark because it is readily identified, and the MMB usually crosses it superficially. The Hayes-Martin manoeuvre uses this relationship: the vein is ligated inferiorly over the submandibular gland, and the superior stump is retracted upwards with the skin flap, rolling the MMB away from the operative field [35]. The manoeuvre is not oncologically risk-free because perifacial submandibular lymph nodes in contact with the MMB may remain undissected in up to 59% of cases [36].

### 5.4. The Facial Artery Crossing Point as a Landmark

The FA crossing at the IBM is the most reliable landmark for MMB identification [9,37]. Balagopal et al. (2012) reported a mean distance of 1.73 mm below the mandible (SD 1.57 mm) from the IBM to the MMB at this crossing, with the lowest branch 8 mm below and the highest 5 mm above [9]. Baur et al. (2014) found the nerve, on average, 3.6 mm superior to this crossing and 10.9 mm above the IBM at the labial commissure [25]. In an intraoperative series of 85 nerves from 52 otolaryngology patients, Al-Qahtani et al. (2015) similarly identified the point where the FA crosses the IBM as the site at which the MMB was first reliably located during neck dissection, corroborating its value as a primary operative landmark [38].

## 6. Branching Patterns and Their Influence on Course

The number of MMB branches varies between studies. Marcuzzo et al. (2020) reported pooled prevalences of 35% for one branch (95% CI: 18–54%), 35% for two branches (18–54%), 18% for three branches (0–35%), and 2% for four branches (0–8%) [13]. Gatti et al. (2025) [27], pooling 511 hemifaces, found one branch in 56.75%, two in 31.31%, three in 10.57%, and four in 1.37%. Balagopal et al. (2012) [9] found a single division in 79.7% (161/202) of surgical patients, indicating that results depend on the definitions and observation points used [9,27].

Anatomical series show similar variability. Karapinar et al. (2013) found one branch in 36.4% and two in 63.6% [18]. Dar et al. (2018) reported one branch in 28%, two in 52%, three in 18%, and four in 2% at the parotid exit [39]. Das and Bhattacharjee (2024) reported a single MMB in 54.7%, two in 38.7%, three in 5.3%, and four in 1.3% [5]. As noted by Chatterjee (2010), a single-nerve model is misleading: more than one ramus may be present, and the rami may lie at different vertical levels [40]. Table 3 summarises branching prevalence across these series.

The number of branches also affects the vertical course. Gatti et al. (2025) [27] reported that, in two-branch specimens, 68.3% (41/60) of individual rami were below the IBM and 31.7% above. In three-branch specimens, 46.7% were below and 53.3% above [27]. Thus, a higher branch count increases the likelihood that at least one ramus will course below the mandibular margin [44,45]. At its origin, the MMB most often begins as a single branch (56–88%) and then ramifies toward its muscular terminations [1].

## 7. Anastomotic Communications

**Figure 2:**
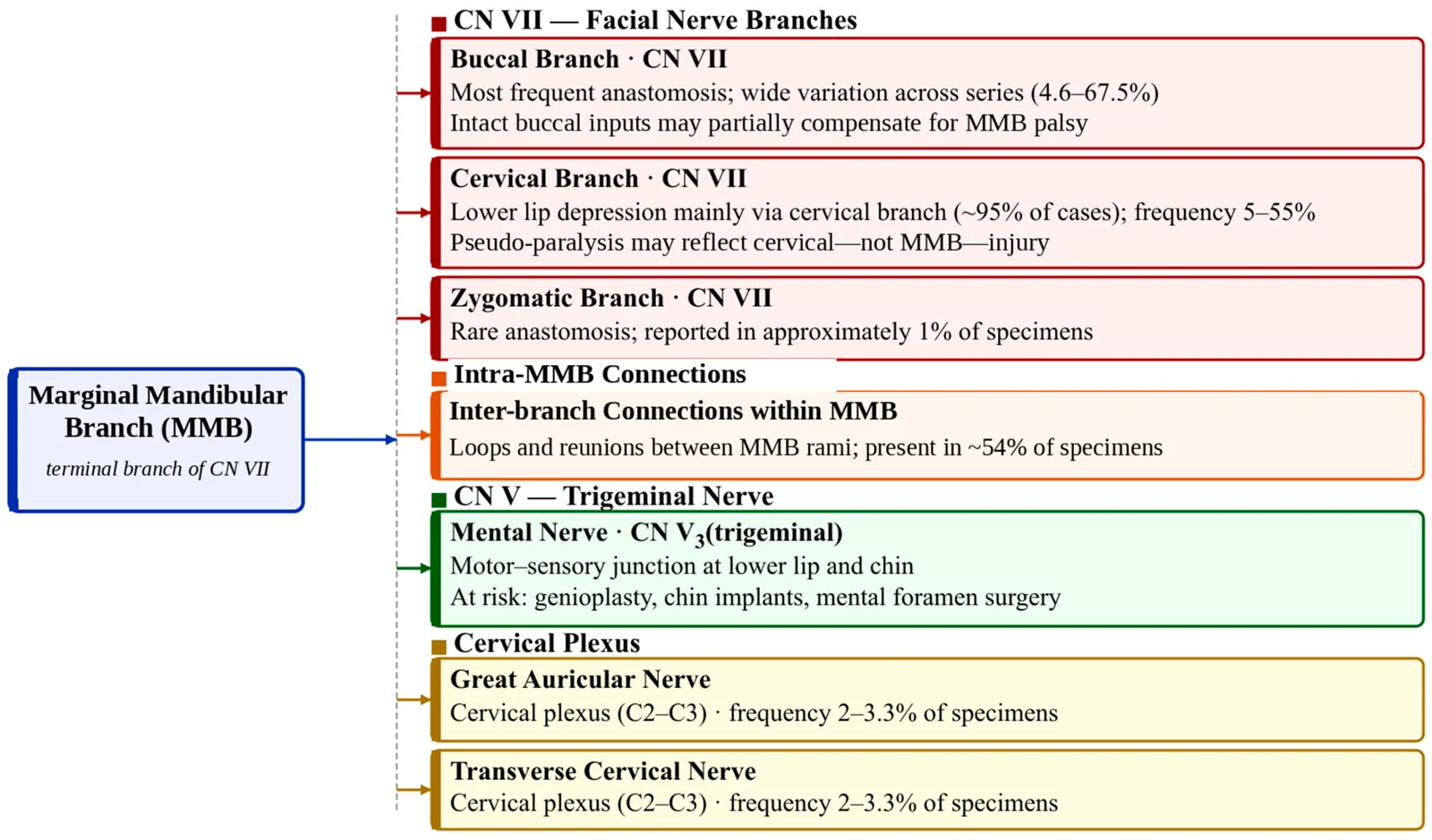
Anastomotic communications of the marginal mandibular branch of the facial nerve: a schematic overview. The MMB (central node, ultramarine) is connected via a dashed communication bus to seven communicating structures arranged in four colour-coded groups: branches of the facial nerve—CN VII (dark red), comprising the buccal, cervical, and zygomatic branches; intra-MMB inter-branch connections (orange); the mental branch of the mandibular division of the trigeminal nerve—CN V_3_ (dark green); and branches of the cervical plexus (yellow), comprising the great auricular and transverse cervical nerves. The left accent bar and border of each node reflect the group colour; box fill represents the lightest tint of that colour. Arrows indicate the direction of communication from the MMB. Each node includes key frequency ranges and principal clinical implications. CN = cranial nerve; MMB = marginal mandibular branch of the facial nerve.

The MMB often communicates with other nerves (Figure 2). The commonest communication is with the buccal branch of the facial nerve. Still, reported frequency differs widely: 44.65% in a pooled analysis of 215 MMBs [27], 40% in Khanfour and El-Sayed (2014) [16], 42.22% in Woltmann et al. (2006) [37], and 4.6% in Karapinar et al. (2013) [18]. Other series reported frequencies of 12–20% [5,19]. These frequencies are strongly method-dependent: standard dissection clusters at the lower end (4.6–20%, including the 20% pooled by Marcuzzo et al.), whereas high-magnification microdissection yields substantially higher values (up to 67.5%; Section 13), so the apparent discrepancy reflects detection sensitivity rather than true anatomical variation.

Freed et al. (2022) mapped the communicating facial nerve branches in anatomical hemifaces. They found buccal-MMB connections in 67.5% and MMB-cervical connections in 55%, both higher than in most earlier series [43]. Toure et al. (2019) also found frequent inter-branch communications and noted that the MMB is more consistently lateral to the FV than to the FA [41]. These buccal, marginal mandibular, and cervical connections help explain variable deficits and occasional spontaneous recovery after isolated MMB injury [41,42,43].

Additional anastomoses include the cervical branch of the facial nerve (5–22.22%), the mental nerve (6.51–28%), the anterior branch of the great auricular nerve (2–3.3%), the transverse cervical nerve (2–3.3%), and, rarely, the zygomatic branch (1%) [5,13,16,27,37]. Communications can also occur within the MMB itself: Khanfour and El-Sayed (2014) reported inter-branch communications in 53.6% of specimens [16]. Balagopal et al. (2012) identified MMB–cervical branch communication in 49 of their 202 patients [9].

The buccal-marginal anastomosis may partly compensate for isolated MMB injury. Conversely, the MMB has been described as the facial nerve branch least likely to recover spontaneously because it has fewer anastomotic connections than other branches [19]. This traditional characterisation rests on standard dissection; higher-magnification microdissection reveals a substantially denser communicating network (Section 13) that better accounts for the partial, variable deficits and the spontaneous recovery frequently seen after isolated MMB injury.

### MMB–Mental Nerve Anastomoses

The anastomosis between the MMB, a motor branch of CN VII, and the mental nerve, a sensory branch of the inferior alveolar nerve, has additional surgical relevance. These motor-sensory connections form a network around the lower lip and chin, creating both a risk zone and a potential route for compensation after lower facial nerve injury. Minelli et al. (2023a), in anatomical work on the platysma and lower lip, found MMB-to-mental nerve anastomosis in all dissected specimens [46]. Tzafetta and Terzis (2010) also documented distal connections between marginal mandibular branches and the mental nerve, as well as buccal and infraorbital connections [42]. Toure et al. (2019) and Robinson et al. (2020) reported frequent connections with trigeminal branches [41,47].

Terminal MMB branches to the platysma, DLI, DAO, and mentalis converge with branches of the mental nerve, forming a sensory-motor unit for the lower lip [46,48]. This network may preserve some movement or sensation after isolated MMB or mental nerve injury [46,47,48]. In lower-lip surgery, genioplasty, chin implantation, or mental nerve decompression, injury near the mental foramen can therefore affect both sensation and mimetic control [41,42,47]. Table 4 summarises reported communicating branch frequencies across these mapping studies.

## 8. Three General Pathways of the MMB

**Figure 3:**
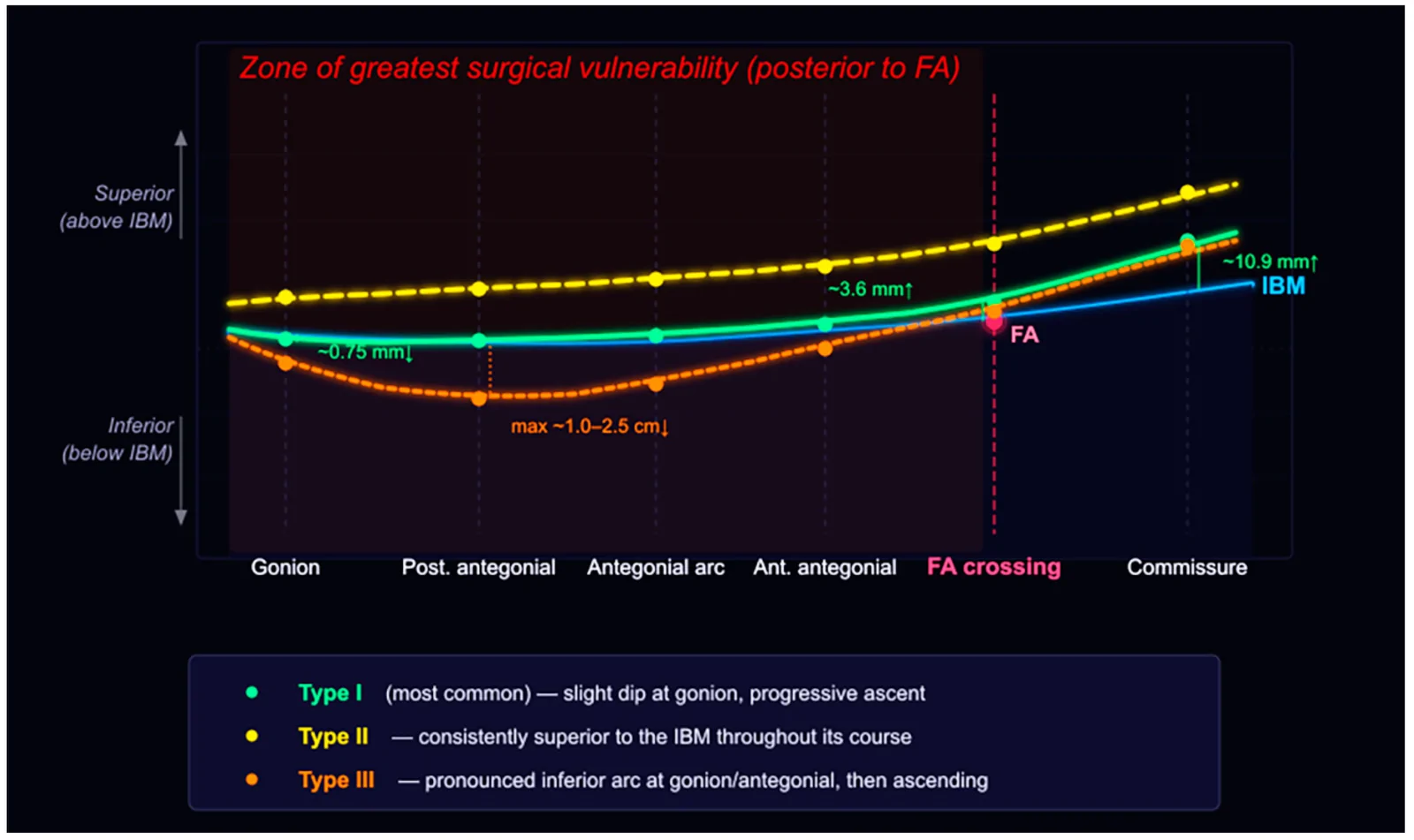
Schematic representation of the three general pathways of the marginal mandibular branch of the facial nerve (MMB) in relation to the inferior border of the mandible (IBM) and key landmarks. Type I (green, solid): slight dip at the gonion, followed by anterior ascent. Type II (blue, dashed): course consistently superior to the IBM. Type III (orange, dotted): pronounced inferior arc in the gonion/antegonial region, followed by anterior ascent. The red circle marks the facial artery (FA) crossing on the IBM. The shaded zone indicates the most vulnerable segment, posterior to the FA. Based on Baur et al. (2014) [25].

Baur et al. (2014) classified the MMB into three general pathways using five mandibular landmarks: the gonion, the posterior and anterior borders of the antegonial notch (AGN), the antegonial arc, and the FA-IBM crossing [25]. Their anatomical measurements supported three patterns (Figure 3, Table 5):

**Table 5:** Three general pathways of the marginal mandibular branch of the facial nerve (MMB) relative to the inferior border of the mandible (IBM), as proposed by Baur et al. (2014) [25]. Distances are averaged anatomical measurements.

Pathway	Description
Type I (most common)	The nerve dips slightly below the gonion (~0.75 mm), then courses along or just above the IBM through the antegonial region, ascending progressively to ~3.6 mm above the IBM at the facial artery crossing and ~10.9 mm above at the labial commissure.
Type II	The nerve consistently runs superior to the IBM throughout its course, never dipping below the mandibular margin. This represents the ‘classical’ textbook description.
Type III	The nerve forms a more pronounced inferior arc, as depicted in Figure 4, dipping significantly below the IBM at the gonion and antegonial region (up to 1.0–2.5 cm) before ascending anteriorly to cross the facial artery above or at the level of the IBM.

**Figure 4:**
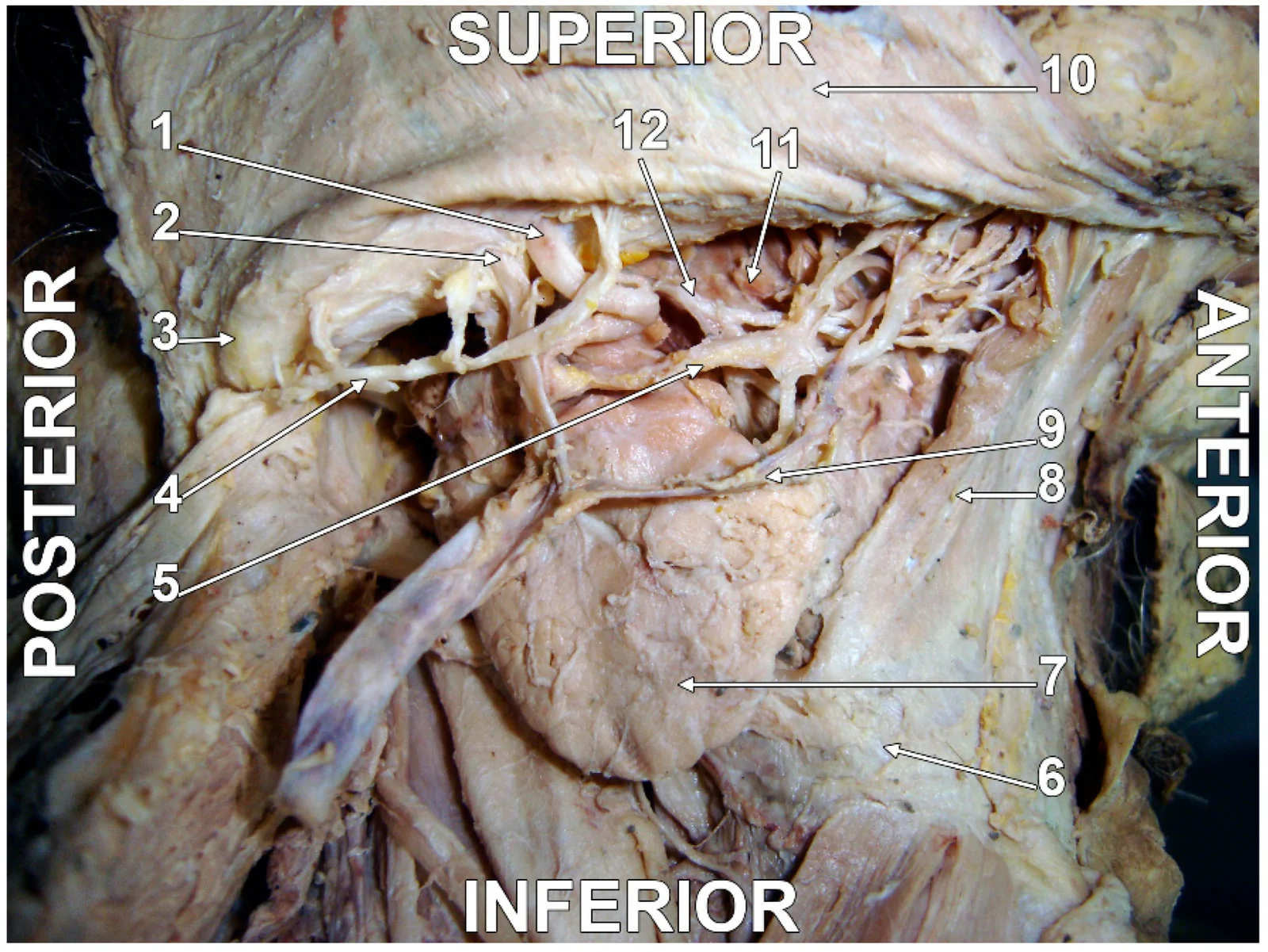
Dissection of the right submandibular region, lateral view. Type III (pronounced inferior arc) of the marginal mandibular branch of the facial nerve (MMB). 1. facial artery; 2. facial vein; 3. gonial angle; 4. MMB; 5. submental artery; 6. hyoid body; 7. submandibular gland; 8. anterior belly of the digastric muscle; 9. submental vein; 10. platysma muscle (elevated); 11. mylohyoid muscle; 12. mylohyoid nerve.

The classification provides a practical operative model and identifies the highest-risk segment between the parotid gland and the facial vessels, particularly the gonion-to-antegonial region, where the nerve may reach its lowest position.

The AGN is not a constant landmark. Tunis et al. (2025), using computed tomography scans of 311 adults, found that the AGN was absent in about one-third of adults: 22.7% of males and 35.1% of females [50]. When present, it was sexually dimorphic, with males having more than twice the AGN area of females (55 mm^2^ vs. 31 mm^2^; *p* < 0.001), even after adjustment for mandibular size [50]. Presence, but not size, was associated with facial growth direction: absent AGN was most frequent in short-face individuals and least frequent in long-face individuals. AGN size was not associated with masticatory muscle dimensions. Surgically, these findings indicate that the AGN cannot be used alone to predict MMB position; alternative bony and vascular landmarks should be used when the AGN is absent or poorly defined [50].

## 9. Functional Innervation of Lower Lip Target Muscles

**Figure 5:**
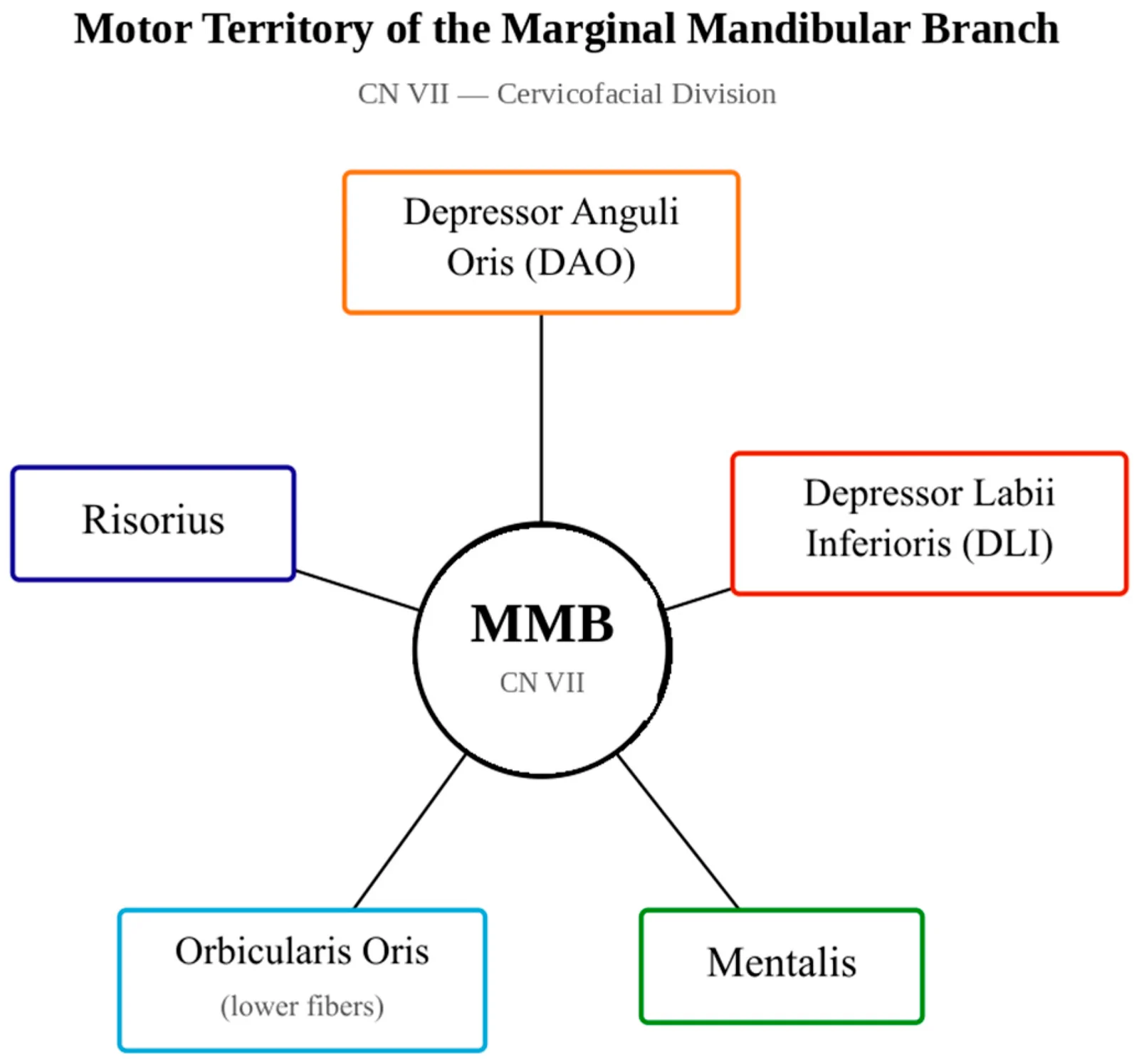
Muscular territory of the marginal mandibular branch of the facial nerve.

Recent functional studies refine the classical description of lower-lip innervation. Intraoperative electrostimulation shows that there is no fixed one-branch-one-muscle pattern: several branches can produce the same movement, and lower facial muscles receive overlapping inputs [51]. The MMB reliably supplies the DAO, lower orbicularis oris, mentalis, and facial platysma (Figure 5). However, Kaufman-Goldberg et al. (2023) and Har-Shai et al. (2026) showed that lower-lip depression and smile-associated dental display are driven mainly by the cervical branch rather than the MMB in approximately 95% of cases [48,52]. These findings influence how postoperative deficits are interpreted and how selective nerve transfers are planned.

The DAO and mentalis receive buccal, marginal mandibular, and cervical inputs [46,48,52]. The platysma is supplied along its deep surface by the cervical branch, usually via a single main cervical trunk [46,52,53]. Overlapping innervation, together with the communicating branch network (Table 4), explains why isolated MMB injury may produce partial or variable deficits and why recovery may occur through intact buccal and cervical branches. Table 6 summarizes the muscle-specific innervation patterns described above.

## 10. Anatomical Versus Intraoperative Observations

Anatomical and intraoperative studies report different MMB levels. In vivo studies often report a higher position than anatomical studies. Al-Qahtani et al. (2015), in 85 nerves from 52 otolaryngology patients, found the mean position to be 0.2–3.4 mm above the mandibular margin [38]. Baker and Conley (1979) and Nelson and Gingrass (1979), from operative experience, argued that the nerve lies below the mandibular border in nearly all surgical cases [29,54].

Neck position may partly explain this difference. Extension and contralateral rotation tension the investing (superficial) layer of the cervical fascia and displace the nerve. Nason et al. (2007) measured the lowest point at 1.25 ± 0.7 cm below the mandible during neck extension [6]. Marolt et al. (2021) [26] compared fresh and embalmed anatomical specimens and found no significant difference in MMB position, indicating that tissue fixation is not the sole explanation. In 62 anatomical hemiheads and 6 fresh head and neck sides, with the neck extended to 15 degrees to simulate surgical positioning, they measured the most inferior MMB branch relative to the AGN: the nerve was below the mandibular border in 90.3% of specimens and above it in 9.6%, with a mean of 4.22 mm below the notch (range: −11.71 to 12.06 mm). There were no significant differences by fixation status (3.78 vs. 4.37 mm; *p* = 0.433), sex (*p* = 0.835), side (*p* = 0.725), or age (*p* = 0.564) [26]. Across all specimen types, the nerve lay within 1 cm of both the gonial angle and the AGN; in the six fresh head and neck specimens used as a supplementary comparison, the mean distance from the MMB to the gonial angle was 0.8 ± 2.07 mm below it [26]. The authors recommended incisions at least 3 cm below the mandibular border [26]. Sindel et al. (2021) similarly found, in 12 fresh anatomical specimens, that the nerve was most often above the mandibular base but could descend 4 mm below it, with significant left-right asymmetry in the middle part of the course [23].

## 11. Surgical Injury: Incidence, Mechanisms, and Recovery

### 11.1. Incidence by Procedure

Reported MMB injury rates vary by operation and by definition of palsy. Kudva et al. (2021) listed rates of 0–20% after submandibular gland excision, 5.6–16.3% after parotidectomy, and up to 23% after neck dissection [8]. Dissection around the nerve during neck clearance leads to temporary neuropraxia in 16–23% of cases [6,55]. In a clinical series of 202 neck dissections with the nerve identified and preserved, Balagopal et al. (2012) found temporary dysfunction in 39 patients (19%), consistent with this range; nearly all recovered [9]. Immediate postoperative marginal mandibular weakness has been reported in up to 28.7% of cases, with persistent palsy up to 16% [19].

Transient deficit rates are higher after some procedures. Babuci et al. (2025) [4] attributed transient paresis after parotid ablation to excessive traction and microtrauma in 47.1% of cases. In the submandibular triangle, transient paresis was reported in 29.8% of cases, usually after retractor use [4]. After facelift surgery, MMB injury is more likely to be symptomatic than injury to other facial nerve branches [56]. Daane and Owsley (2003) [56] also distinguished true MMB injury from ‘MMB pseudo-paralysis’, in which the observed lower-lip asymmetry results from injury to the cervical branch rather than the MMB itself; this distinction is clinically relevant because cervical branch injuries tend to recover more completely. The anatomical basis for this distinction is reinforced by the simple branching architecture of the cervical branch—a single trunk with no proximal ramification targeting exclusively the platysma—whose disruption during necklift or platysmaplasty may produce recurrent platysma bands mimicking lower lip weakness [57].

### 11.2. Risk Factors and Mechanisms

Anatomical variability is a major risk factor. Multiple branches increase the probability that the lowermost ramus lies below the IBM [44]. Another vulnerable site is the area over and anterior to the masseter, where the nerve is superficial, beneath a thin SMAS layer [19]. The MMB has been described as the facial nerve branch least likely to recover because it communicates less often with other branches. However, microanatomical evidence of richer anastomoses now tempers this classical view (Section 13) [19]. Parotid and submandibular operations remain the principal surgical settings for iatrogenic injury.

### 11.3. Recovery and Repair

Prognosis depends on the type of injury. In axonotmesis, with the endoneurium intact, fibres regenerate at about 1 mm per day. In neurotmesis, complete transection requires repair within two to six weeks [19]. If transection is recognised intraoperatively, immediate neurorrhaphy with 8-0 to 10-0 monofilament nylon sutures in the epineurium under magnification is recommended [58]. Balagopal et al. (2012) reported temporary dysfunction in 39 of 202 patients (19%), with recovery in nearly all cases [9].

## 12. Clinical Implications for Surgical Safety

### 12.1. Incision Placement

Incision recommendations vary with study population, anatomical definition, and accepted risk threshold. Dingman and Grabb’s (1962) 2 cm rule [12] has been widely used but is not protective in all cases. Davies et al. (2016) found the most inferior MMB branch below the 2 cm line in 7 of 31 specimens and proposed a two-finger-breadth landmark (25–51 mm) as a safer alternative [59]. Aravena et al. (2014) considered 1 cm below the mandible safe in their population, whereas Woltmann et al. (2006) recommended 3 cm below the mandible with careful plane dissection and flap retraction [37,60]. Gündoğdu et al. (2024) cautioned that incisions 2 cm below the IBM may still endanger the nerve when neck extension displaces it inferiorly [28].

### 12.2. Safe Dissection Planes

Posterior to the facial vessels, a subplatysmal plane superficial to the cervical fascia leaves a protective tissue bridge over the nerve. Anterior to the vessels, a supraperiosteal plane along the mandibular margin beneath the platysma is recommended [7,8]. Maintaining the correct platysma-SMAS plane reduces the risk of injury [8]. Preserve all visible MMB branches, especially when multiple rami are present [9].

### 12.3. The Hayes-Martin Manoeuvre

The Hayes-Martin manoeuvre consists of ligating the FV about two finger breadths below the mandible and retracting the superior stump with the skin flap, moving the MMB superiorly and away from the operative field. It is useful for nerve preservation, but it may reduce clearance of perifacial level IB lymph nodes; its use should therefore be adapted to the oncological context [35,36].

### 12.4. Implications for Rhytidectomy and the Mandibular Ligament

In facelift surgery, mandibular ligament release may be used to correct the jowl. Minelli et al. (2023b) [22] showed that the true mandibular ligament exists only in the deep subplatysmal plane and has no definite subcutaneous osteocutaneous component. The jowl forms in the subcutaneous layer over the posterior part of this deep ligament, and the main MMB branch passes only 1–2 mm from it. Deep release of the ligament therefore places the MMB at risk and is not recommended. In subcutaneous-plane rhytidectomy, the released structure is the connection between the anterior jowl dermis and the musculoligamentous attachment of the platysma, DLI, and DAO. A deep-plane, subplatysmal lower facelift can improve the jowl by tightening platysma laxity above its mandibular attachment and transmitting that tension through the retinacula cutis, without directly exposing the MMB [22].

### 12.5. Postoperative Assessment and Monitoring

MMB dysfunction is assessed primarily by clinical examination. Electromyography and nerve conduction studies can define the injury site and severity and help distinguish peripheral palsy from central lesions [19]. Routine intraoperative nerve monitoring is uncommon during submandibular gland excision, but handheld monitoring without intravenous paralytics may be useful when malignancy or severe chronic infection obscures the surgical planes [58].

## 13. Conclusions

The evidence reviewed here supports a segment-based rather than a fixed-distance model of MMB surgical anatomy. Risk depends on branching pattern, relation to the facial vessels, fascial plane, and operative positioning. Accordingly, neither the IBM nor any single incision-distance rule should be treated as an independent predictor of nerve position.

Position, vascular relations, and pathway. At least one MMB branch lies at or below the IBM in 39% of hemifaces in the pooled estimate of Marcuzzo et al. (2020) and in up to 70% in Gatti et al. (2025) [13,27]. These estimates differ methodologically, but both show that an inframandibular branch is too common to support a universally safe distance from the mandibular border. The segment posterior to the FA is the most variable. The MMB crosses the FV superficially in the great majority of specimens and the FA superficially in most, although deep, mixed, and looping configurations occur. The premasseteric branch adds a vascular hazard: its origin is approximately 11.25 mm above the IBM and its reported separation from the MMB can narrow to 3 mm [34]. Baur et al. (2014) organise this variability into three trajectories, from a course consistently above the IBM to a pronounced inferior arc [25], while the needle-mapped data of Sindel et al. (2021) place much of the nerve within a narrow corridor around the mandibular margin [23]. These observations favour recognising spatial trajectory and vascular relationships rather than relying on a single linear landmark.

Fascial planes and landmarks. The nerve’s relationship to superficial tissues changes along its course. Posteriorly, subplatysmal dissection superficial to the cervical fascia leaves a protective tissue bridge; near the mandibular margin, transition into the platysma-SMAS plane increases vulnerability during superficial dissection. This plane change is as important as distance from the IBM. Bony landmarks are also imperfect when used alone. The AGN is absent in approximately one-third of adults and varies with sex and craniofacial morphology [50]. The IBM, gonion, FA crossing, FV, and local fascial plane should therefore be interpreted together rather than as competing single landmarks.

Communications and functional redundancy. The classical impression that the MMB has few anastomoses depends strongly on the dissection method. Marcuzzo et al. (2020) reported a pooled MMB-buccal communication frequency of about 20% [13]. In contrast, higher-magnification mapping by Freed et al. (2022) demonstrated buccal-MMB connections in 67.5% and MMB-cervical connections in 55% of hemifaces [43]. Targeted studies likewise identify frequent MMB-mental nerve communications [42,46], well above the 12% pooled estimate reported by Marcuzzo et al. (2020) [13]. This richer network is functionally relevant. Current electrostimulation and anatomical evidence indicates that lower-lip movement is not organised as a one-branch-one-muscle system: the DLI is predominantly supplied by the cervical branch, whereas the DAO and mentalis receive convergent inputs from buccal, marginal mandibular, and cervical branches [48,52]. These redundancies provide a plausible substrate for partial deficits and spontaneous recovery after an apparently isolated MMB injury and caution against diagnosing MMB dysfunction solely from lower-lip asymmetry.

Operative implications. The true mandibular ligament is located in the deep subplatysmal plane, and the main MMB branch may pass only 1–2 mm from it [22]; consequently, deep ligament release during rhytidectomy can place the nerve directly at risk. Iatrogenic dysfunction is nevertheless often transient: neck-dissection series report neuropraxia in approximately 16–23% of patients [6,55], and recovery is common when continuity is preserved. The practical objective should therefore be to prevent traction, compression, thermal injury, and transection rather than rely on the expectation of recovery. Because branch number, course, and interconnections vary between individuals, all visible rami should be regarded as potentially functional and preserved whenever possible.

In summary, the MMB is better understood as a variable plexiform motor system than as a single nerve following a predictable line. The most consistent protective strategy is triangulation: combine the IBM and gonion with the FA and vein, anticipate the premasseteric arterial branch, account for neck position and fascial transitions, and remain in the appropriate subplatysmal or supraperiosteal plane. This model accommodates anatomical, microanatomical, and operative observations without converting one measurement into an absolute rule. Safe exposure in the submandibular, parotid, masseteric, cervicofacial, and lower-lip regions therefore depends on recognising local relationships rather than applying a fixed distance from the mandible.

## Figures and Tables

**Table 1:** Summary of key studies reporting the position of the marginal mandibular branch of the facial nerve relative to the inferior border of the mandible (IBM). Values marked with an asterisk (*) refer to the posterior-to-facial-artery segment. Studies marked with a dagger (†) are systematic reviews or meta-analyses. PP = pooled prevalence; FA = facial artery.

Study	n	Above IBM (%)	Below IBM (%)	Max. Distance Below IBM
Dingman and Grabb (1962) [12]	100	81% *	19% *	1.0 cm
Wang et al. (1991) [24]	120	67% *	33% *	−
Batra et al. (2010) [1]	50	52% (along) + 16% (above) *	32% *	1.6 cm (angle); 1.4 cm (body)
Balagopal et al. (2012) [9]	202	−	−	0.8 cm at FA crossing
Karapinar et al. (2013) [18]	44	100%	0	− (100% above IBM; mean 21.9 mm above)
Baur et al. (2014) [25]	anatomical specimens	−	−	3 pathways identified
Anthony et al. (2018) [15]	anatomical specimens	−	−	Mean max 7.12 ± 2.97 mm
Marcuzzo et al. (2020) † [13]	1861	61%	39% (PP)	−
Marolt et al. (2021) [26]	62	9.6%	90.3%	−
Sindel et al. (2021) [23]	24	Highest prevalence	−	Max 4 mm (R), 3 mm (L); needle-mapped pathway
Gatti et al. (2025) † [27]	511	30%	70%	Mean 2.53 cm
Gündoğdu et al. (2024) [28]	96	−	−	mean 2.57 mm (R)/3.53 mm (L); 80% below IBM; max ≤ 10 mm (all but 3 necks)

**Table 2:** Reported relationship of the marginal mandibular branch of the facial nerve (MMB) to the facial vessels across selected studies. FA = facial artery; FV = facial vein.

Study	n	Relationship to Facial Vessels
Khanfour and El-Sayed (2014) [16]	30 hemifaces	83.3% superficial (lateral) to both FA and FV
Karapinar et al. (2013) [18]	44 hemifaces	97.7% lateral to FA; 1 specimen between FA and FV
Basar et al. (1997) [17]	anatomical specimens	42% superficial to FA; 4% deep; 54% both sides
Babuci et al. (2025) [4]	59 rami	93.2% crossed externally; 6.8% looped/reunited around vessels
Marcuzzo et al. (2020) [13]	1861 (pooled)	PP 38% lateral to FV (single); 57% lateral (multiple)
Batra et al. (2010) [1]	50 hemifaces	100% superficial to FA and FV
Savary et al. (1997) [32]	anatomical specimens	Neural plexus around FA; some branches deep to FA, superficial to the vein
Touré et al. (2004) [33]	54 dissections	94% (51/54) lateral to facial vessels; 2 medial to artery, lateral to vein; 1 lateral to artery, medial to vein

**Table 3:** Branching prevalence of the marginal mandibular branch of the facial nerve (MMB) across representative anatomical and clinical series.

Study Context	Branch Counts	Key Numeric Findings
Marcuzzo et al. (2020) [pooled, 1861] [13]	1–4	1: 35%; 2: 35%; 3: 18%; 4: 2%
Gatti et al. (2025) [pooled, 511] [27]	1–4	1: 56.75%; 2: 31.31%; 3: 10.57%; 4: 1.37%
Balagopal et al. (2012) [surgical, 202] [9]	1–4	Single: 79.7%; 2: 12.9%; 3: 6.9%; 4: 0.5%
Karapinar et al. (2013) [anatomical, 44] [18]	1–2	1: 36.4%; 2: 63.6%
Dar et al. (2018) [anatomical, parotid exit] [39]	1–4	1: 28%; 2: 52%; 3: 18%; 4: 2%
Das and Bhattacharjee (2024) [anatomical] [5]	1–4	1: 54.7%; 2: 38.7%; 3: 5.3%; 4: 1.3%
Touré et al. (2019) [anatomical] [41]	1–≥4	1: 22.6%; 2: 29%; 3: 12.9%; plexus ≥ 4: 35.5%
Tzafetta and Terzis (2010) [microanatomy] [42]	Mean 2.3	Mean 2.3 branches; buccal–MMB interconnections in 50%
Freed et al. (2022) [anatomical, communicating br.] [43]	–	Buccal–MMB in 67.5%; MMB–cervical in 55% of hemifaces

**Table 4:** Summary of communicating branch frequencies for the marginal mandibular branch of the facial nerve (MMB), compiled from anatomical mapping studies. Values represent the percentage of hemifaces in which the specified communication was identified.

Communication	Frequency	Source(s)
Buccal–MMB	50–67.5%	[42,43]
MMB–cervical	55%	[43]
MMB–mental nerve	100% (in targeted studies)	[42,46]
Temporal–zygomatic	>50–60%	[42,49]
Zygomatic–buccal	>50–60%	[42,43,49]

**Table 6:** Innervation of lower facial muscles by specific facial nerve branches. DAO = depressor anguli oris; DLI = depressor labii inferioris; MMB = marginal mandibular branch of the facial nerve.

Muscle	Predominant Branch(es)	Key Source(s)
DAO	MMB, buccal, cervical (poly-innervated)	[48,52]
DLI	Cervical branch (≈95%); minor MMB contribution	[46,48]
Mentalis	MMB, buccal, cervical (poly-innervated)	[51,52]
Lower orbicularis oris	MMB (primary), buccal connections	[43,51]
Platysma (facial segment)	MMB + cervical (deep surface)	[46,52,53]

## Data Availability

No new data were created or analysed in this study. Data sharing does not apply to this article.

## Abbreviations

The following abbreviations are used in this manuscript:

FV	Facial vein
AGN	Antegonial notch
CI	Confidence interval
CN V_3_	Mandibular division of the trigeminal nerve
CN VII	Facial nerve (cranial nerve VII)
DAO	Depressor anguli oris
DLI	Depressor labii inferioris
FA	Facial artery
IBM	Inferior border of the mandible
MMB	Marginal mandibular branch of the facial nerve
PP	Pooled prevalence
SD	Standard deviation
SMAS	Superficial musculo-aponeurotic system
